# Energy-Dense High-Fat/High-Sucrose Diet to Induce Type 2 Diabetes Mellitus in BALB/c Mice Without Genetic Modifications and Chemical Agents

**DOI:** 10.3390/biology15020109

**Published:** 2026-01-06

**Authors:** Alma Nelly Diaz-Herreros, Amaranta Sarai Valdez-Guerrero, Juan Carlos Cancino-Díaz, Luvia Enid Sánchez-Torres, Fernando Gómez-Chávez, Mónica Gricelda Arellano-Mendoza, Feliciano Tamay-Cach, Mario Eugenio Cancino-Diaz

**Affiliations:** 1 Laboratorio de Inmunología Aplicada, Departamento de Inmunología, Escuela Nacional de Ciencias Biológicas del Instituto Politécnico Nacional, Prolongación de Carpio y Plan de Ayala s/n, Col. Santo Tomas, Delg. Miguel Hidalgo, Mexico City 11340, Mexico; ndiazh1917@alumno.ipn.mx; 2 Centro de Investigación Científica Serendipia A.C., Tláhuac, Mexico City 13210, Mexico; 3 Laboratorio de Bioquímica Aplicada, Escuela Superior de Medicina del Instituto Politécnico Nacional, Mexico City 11340, Mexico; 4 Laboratorio de Inmunología y Microbiología, Departamento de Microbiología, Escuela Nacional de Ciencias Biológicas del Instituto Politécnico Nacional, Prolongación de Carpio y Plan de Ayala s/n, Col. Santo Tomas, Delg. Miguel Hidalgo, Mexico City 11340, Mexico; 5 Laboratorio de Inmunología de los Microorganismos, Departamento de Inmunología, Escuela Nacional de Ciencias Biológicas del Instituto Politécnico Nacional, Prolongación de Carpio y Plan de Ayala s/n, Col. Santo Tomas, Delg. Miguel Hidalgo, Mexico City 11340, Mexico; 6 Laboratorio de Enfermedades Osteoarticulares e Inmunológicas, Sección de Estudios de Posgrado e Investigación, Escuela Nacional de Medicina y Homeopatia del Instituto Politécnico Nacional, Av. Guillermo Massieu Helguera 239, Mexico City 07320, Mexico; 7 Laboratorio de Enfermedades Crónico Degenerativas, Sección de Estudios de Posgrado e Investigación, Escuela Superior de Medicina del Instituto Politécnico Nacional, Mexico City 11340, Mexico

**Keywords:** type 2 diabetes mellitus, high-calorie diet, animal model, BALB/c mice, C57BL/6 mice

## Abstract

Type 2 diabetes mellitus (T2DM) is a highly prevalent disease characterized by chronic hyperglycemia, commonly associated with intake of a high-calorie diet (HCD). Although numerous T2DM murine models have been developed using C57BL/6 mice, BALB/c mice typically fail to develop the disease under the same conditions. We hypothesized that diets optimized for C57BL/6 mice may be insufficient to induce T2DM in BALB/c mice. Female BALB/c and C57BL/6 mice (*n* = 48 each) were fed either a specific high-calorie diet (HCD) or a standard diet (SD) for ten weeks. BALB/c mice fed a specific HCD exhibited developed persistent hyperglycemia (112.6 mg/dL ± 3.4) from week 1 through week 10, while SD-fed controls maintained normal glucose levels (84.2 mg/dL ± 2.8). HCD-fed BALB/c mice showed elevated serum insulin (39.09 pg/dL ± 25.94), triglycerides (290.8 mg/dL ± 139.5), HOMA index (7.68 ± 1.49) and high post-challenge glucose along with visceral adiposity, hepatic steatosis, and pancreatic alterations. SD-fed BALB/c mice showed no such changes. Similar findings were observed in C57BL/6 mice, used as a positive disease-control group. This model demonstrates that a properly formulated HCD can induce T2DM in BALB/c mice, enabling the study of genes and molecules associated with diabetes susceptibility without requiring genetic or chemical manipulation.

## Simple Summary

BALB/c mice usually do not develop type 2 diabetes mellitus (T2DM) when fed commercial hypercaloric diets. In this study, we demonstrate that a laboratory-formulated, energy-dense diet rich in fat and sucrose successfully induces T2DM in BALB/c mice. After ten weeks on this diet, BALB/c mice exhibited hallmarks of T2DM, similar to C57BL/6 mice. These findings indicate that the caloric density of the diet may be more critical than genetic background for the development of T2DM.

## 1. Introduction

Diabetes mellitus is a metabolic disorder characterized by a chronic elevation in blood glucose levels, resulting from inadequate insulin production, defective insulin signaling, and/or impaired glucose metabolism. When diabetes is not controlled in humans, several complications can occur in different organs, such as diabetic nephropathy, diabetic retinopathy, diabetic neuropathy, and non-alcoholic fatty liver disease. Other complications include hearing impairment, foot damage, cardiovascular disease (heart attack, stroke), and others. The global prevalence of diabetes for all age groups was 2.8% in 2000 and is projected to reach 4.4% in 2030, suggesting that by 2030, approximately 366 million people worldwide will have diabetes. In urban populations of developing countries, diabetes is more common, especially in people over 65 years old. Diet and sedentary lifestyles are important factors contributing to the development of this disease. Given the global importance of diabetes and the role of high-fat diets in its development, several diet-induced animal models have been generated to study this disease [1].

Various animal models of type 2 diabetes mellitus (T2DM) have been studied using different species, including non-human primates, transgenic pigs, cats, dogs, rats, and mice. While non-human primates provide the closest physiological resemblance to human diabetes, their high maintenance costs limit their practical use. Feline models show several metabolic traits similar to those in human diabetes but are not suitable for large-scale studies. Rodents, especially rats and mice, are preferred for their low cost and ease of genetic manipulation [2].

Genetic models of type 1 diabetes in rodents include BioBreeding rats (BB rats), which spontaneously develop type 1 diabetes associated with autoimmune reactions linked to MHC genes and a mutation in the gimap5 gene. They exhibit severe lymphopenia and immunocompromised conditions [3,4]. LEW 1AR1/-iddm rats were the first rat model to spontaneously undergo autoimmune destruction of its islet beta cells. Rats present a spontaneous mutation associated with the telomeric region of rat chromosome 1 (Iddm8) in a colony of LEWIS.1AR1 rats [5]. Non-obese diabetic (NOD) mice, a polygenic model of T1DM with spontaneous genetic modification, represent another model of diabetes caused by autoimmunity, in which immune cell-driven destruction of pancreatic beta cells is observed [6]. Akita mice carry a mutation in the Ins2 gene that causes misfolding of the insulin 2 protein, leading to pancreatic β-cell death and resulting in hypoinsulinemia and hyperglycemia [7]. Conversely, Zucker diabetic fatty (ZDF) [8] and Goto-Kakizaki (GK) rats are models of T2DM. Zucker fatty rats have a homozygous missense mutation (fatty, Fa) in the gene encoding the rat leptin receptor (Ob-R) [9]. In contrast, GK rats were established by repeated breeding of glucose-intolerant Wistar rats, resulting in a nonobese rat model of Type 2 diabetes [10]. Chemically induced diabetes models, using agents such as streptozotocin (STZ) or alloxan, are also common. Both are cytotoxic glucose analogues that, through GLUT-2, are internalized into beta cells. While STZ fragments beta-cell DNA, alloxan inhibits insulin secretion by blocking glucokinase and also produces reactive oxygen species, leading to beta-cell necrosis [11].

One of the most frequently employed models for T2DM research is the murine model induced by a high-fat diet (HFD) [12,13,14]. According to the United States Centers for Disease Control and Prevention, most T2DM cases in humans are associated with dietary habits rather than genetics, validating the relevance of diet-induced models [15].

Obesity, defined in humans by a body mass index (BMI) over 30 kg/m^2^, often precedes metabolic syndrome, a prediabetic state characterized by obesity, hypertension, hyperglycemia, hypertriglyceridemia, and low HDL cholesterol levels [16,17,18].

Several studies report that most mice subjected to an HFD develop hyperglycemia within 8 to 12 weeks [19]. During this period, these mice often become obese, which frequently precedes metabolic syndrome and leads to visceral obesity and chronic meta-inflammation, ultimately resulting in insulin resistance. Eventually, this process has also been associated with pancreatic beta-cell dysfunction [20,21,22,23,24,25,26]. However, strain-specific responses to HFD have been notably observed. While C57BL/6 mice are prone to T2DM under HFD conditions, strains such as BALB/c, MRL, and A/J are commonly used in research because they do not develop diabetes under the same HFD conditions as C57BL/6. Nonetheless, some studies suggest transient hyperglycemia in BALB/c mice that resolves more quickly than in C57BL/6 mice [27,28].

This study introduces a laboratory-formulated HCD designed to induce T2DM in BALB/c mice. The diabetes-related clinical parameters observed in this strain were like those seen in C57BL/6 mice with diabetes. Inducing T2DM in BALB/c mice, which are less susceptible than C57BL/6, is very important because these models (using HCD only, without chemical agents as in other studies) can be used to investigate new molecules or genes involved in T2DM susceptibility [11] in addition to emulating the natural history of the disease in different genetic backgrounds.

## 2. Materials and Methods

### 2.1. Animal Model and Experimental Design

As our study aimed to investigate whether BALB/c mice can develop T2DM like C57BL/6 mice, a total of 48 BALB/c and 48 C57BL/6 female mice were used, and a specific HCD was formulated for each strain (see Section 2.2: Diet Formulation, Table 1 and Table 2). All mice in the study were aged 4 months, weighed between 21 and 24 g, and were housed in groups of three per cage in a room with a 12 h light/dark cycle. BALB/c mice were randomly divided into two groups: the HCD and SD groups, each with 12 mice. The HCD group was fed laboratory-made HCD croquettes, specific to BALB/C, while the SD group received Standard Diet which was Labdiet 5001 (Purina, Franklin, IN, USA) croquettes for 10 weeks. Similarly, two groups of 12 C57BL/6 mice were also randomly divided. The HCD group was fed specific HCD croquettes for C57BL/6 and the SD group with SD chow croquettes for 10 weeks. C57BL/6 are known for their susceptibility to T2DM, and that is why they were used as the disease-test group. Every experiment was performed twice. Fasting glucose levels were measured weekly. The protocol to HCD-T2DM induction was based on Appiakannan et al. in 2020, reported to C57BL76 [19]. Nevertheless, in previous experiments we assayed both female and male mice to induce T2DM with HCD, and we observed that with our diet the female mice presented higher levels of glucose in serum than the males, and that is why we used only female mice in all the experiments. At the end of the study, animals were euthanized with a lethal dose of pentobarbital (Aranda-Fynsa, Mexico City, Mexico); 150 mg/Kg body weight administered intraperitoneally after 5 h of fasting [29] and following ENCB-IPN ethical guidelines. A blood sample without an anticoagulant to obtain serum was collected by cutting the furthest part of the tail, and the blood was collected by dripping. The serum was used to determine insulin, triglyceride, and cholesterol levels. Visceral adipose tissue, pancreas, and liver were excised, weighed, fixed in 4% formalin (Sigma-Aldrich, St. Louis, MO, USA), and embedded in paraffin for histological analysis, which is helpful to confirm the diagnosis of diabetes [27,30,31].

### 2.2. Diet Formulation

Two experimental HCDs were formulated and prepared in the laboratory. One was designed to induce T2DM in BALB/c mice with higher carbohydrate and fat content, to give a total of 4.89 Kcal/g of solid diet while the other with 4.31 KCal/g of solid diet aimed to induce T2DM in C57BL/6 mice and contained more carbohydrate but less fat than the BALB/c diet. Commercially available chow 5001 LabDiet was used as standard diet (SD) which give 3.35 KCal/g of solid diet. Both HCD included the following ingredients (see Table 1): commercially available chow 5001 LabDiet, wheat flour (as an additional carbohydrate source), eggs (as an extra protein source), and lard (as an extra fat source). Commercial chow croquettes were crushed using a shredder and then sieved. Afterward, they were mixed with wheat flour, eggs, and fat until a homogeneous mixture was achieved. This mixture was then used to produce new mice chow croquettes in small molds, which were stored at −20 °C until use. The mice were fed ad libitum with these croquettes, and to complete the HCD, sucrose was added to their drinking water (30% *w*/*v* for BALB/c, and 20% for C57BL/6). The standard diet (SD) consisted of commercial chow 5001 Lab Diet and sterile water, both of which were provided ad libitum. Diet compositions are detailed in Table 1, and the bromatological analysis is presented in Table 2. Food intake was measured weekly by weighing the amount of food placed in each cage at the start of the week and the remaining food at the end of the week. Food consumption per cage was calculated by subtracting the final food weight from the initial weight. Water intake was measured similarly to food intake.

**Table 1:** Composition of the HCD to BALB/c and C57BL/6 and SD. The mice were fed with a corresponding diet to induce T2DM.

Ingredients of the Diet	HCD to BALB/c	HCD to C57BL/6	SD
Croquettes	1 kg	1 kg	* Lab diet 5001
Wheat flour	300 g	260 g	-
Eggs	6 unit	8 unit	-
Lard	600 g	400 g	-
Sucrose in water	30%	20%	0%
Amount ingested	Ad libitum	Ad libitum	Ad libitum

* Lab diet 5001 composition: Ground Corn, Dehulled Soybean Meal, Dried Plain Beet Pulp, Fish Meal, Ground Oats, Dehydrated Alfalfa Meal, Brewers Dried Yeast, Cane Molasses, Wheat Germ, Dried Whey, Porcine Animal Fat Preserved with BHA and Citric Acid, Porcine Meat and Bone Meal, Wheat Middlings, Salt, Calcium Carbonate, DL-Methionine, Choline Chloride, Cholecalciferol (Vitamin D3), Vitamin A Acetate, Folic Acid, Menadione Dimethylpyrimidinol Bisulfite (Vitamin K), Pyridoxine Hydrochloride, Thiamine Mononitrate, Nicotinic Acid, Calcium Pantothenate, DL-Alpha Tocopheryl Acetate (Vitamin E), Manganous Oxide, Vitamin B12 Supplement, Zinc Oxide, Ferrous Carbonate, Copper Sulfate, Ferrous Sulfate, Riboflavin Supplement, Zinc Sulfate, Calcium Iodate, Cobalt Carbonate, Biotin, Sodium Selenite.

**Table 2:** Bromatological analysis of the new HCD corresponding to each strain and SD. The results are expressed in grams per 100 g of diet.

Components	HCD to BALB/c	HCD to C57BL/6	Standard Diet (SD)
Protein	11.31 ± 0.42	12.63 ± 0.08	23
Carbohydrate	27.02	29.67	25
Lipid	37.3 ± 0.42	29.16 ± 0.19	4.5
Ash	3.76 ± 0.12	4.24 ± 0.11	8
Fiber	6.11 ± 0.08	5.72 ± 0.27	6
Moisture	14.50 ± 0.24	18.58 ± 0.02	12
**Metabolic Energy**			
Calories (Kcal)	489.02	431.64	335
Joules (KJ)	2049.00	1808.57	1401.60

### 2.3. Fasting Blood Glucose and Post-Challenge Glycemia Measurement

Fasting blood glucose and post-challenge glycemia were measured using a glucometer Accu-Chek Instant (Roche Diagnostics, Basel, Switzerland) from a drop of peripheral blood from the tail of mice. Fasting blood glucose levels were checked weekly for 10 weeks. To measure fasting blood glucose, the mice were fasted for 5 h, starting at 8:00 am, and their blood glucose levels were measured afterward. Post-challenge glucose was assayed at 10 weeks in all mice from both strains; to this end, mice were fasted for 5 h, and baseline blood glucose was measured. At the same time, the mice received a load administered through an orogastric cannula at a dose of 2 g/kg body weight of a 50% *w*/*v* sterile D-glucose (Sigma-Aldrich, St. Louis, MO, USA) solution, and blood glucose was measured 45 min after glucose administration. Areas under the curves (AUCs) of post-challenge glucose were calculated by trapezoidal approximation of deviations from the baseline fasting glucose and glucose 45 min post-challenge. The results were presented as AUC (mg/dLx min).

### 2.4. Insulin in Serum Measurement

Insulin serum levels were measured at 10 weeks in all animals in the study using the mouse Ins1 ELISA kit (Sigma-Aldrich, St. Louis, MO, USA). The absorbance was read at 450 nm in a Multiskan FC (Thermo Fisher Scientific, Waltham, MA, USA), and the results are shown in pg/dL.

### 2.5. HOMA-IR Index Determination

HOMA-IR index was calculated using the formula HOMA-IR = fasting glucose (mg/dL) × fasting insulin (μUI/mL)/405.

### 2.6. Serum Measurement of Triglycerides and Cholesterol 

Similarly, during insulin measurement, serum triglycerides and cholesterol were determined at 10 weeks using the Triglycerides (TR8332) and Cholesterol (CH3810) assays from Randox Laboratory, respectively. The results are shown in mg/dL.

### 2.7. Adipose and Pancreatic Index Determination

To determine the adipose index, all visceral adipose tissues were removed and weighed on an analytical balance. The adipose index is the ratio of the weight of removed adipose tissues to the total weight of the mouse. Similarly, the pancreatic index is the ratio of the weight of the pancreas to the total weight of the mouse.

### 2.8. Histological Analysis, Hematoxylin and Eosin Staining

After 10 weeks of feeding, the mice on different diets were euthanized, and the visceral adipose tissue, as well as the pancreas and liver, were collected. The tissues were then placed in a 4% formalin solution for preservation. Following this, the tissues were placed into tissue cassettes and subjected to a dehydration process using an ethanol (Sigma-Aldrich, St. Louis, MO) series (70%, 80%, 90%, 96%, 100%, and 100%) for one hour at each concentration. The tissues were then cleared twice in 100% xylene for one hour per cycle. Subsequently, the tissues were soaked with paraffin at 60˚C for one hour, ensuring complete embedding.

The paraffin-embedded tissues were sectioned at a thickness of 5 µm using a Leica RM 2132 microtome (Leica Microsystems GmbH, Wetzlar, Germany). The tissue sections were placed on slides, which were then immersed in xylene (Sigma-Aldrich, St. Louis, MO, USA) to remove the paraffin. To rehydrate the sections, they were passed through a series of graded ethanol solutions (100%, 96%, and 70%), with each step lasting 2 min, and finally transferred into distilled water.

The rehydrated sections were stained with Hematoxylin and Eosin (H&E) (Sigma-Aldrich, St. Louis, MO, USA). Any excess hematoxylin was removed by differentiating the sections with 15 dips in acid alcohol, which selectively removed excess hematoxylin from the cytoplasm. The slides were then immersed in Scott’s solution for 2 min to neutralize the acid and restore the color of the hematoxylin. Following this, the sections were stained with eosin for 2 min.

After staining, the slides underwent a dehydration process using increasing concentrations of ethanol (80%, 96%, 100%), with 2 min spent in each solution. The alcohol was removed by clearing the slides with xylene. Finally, the tissue sections were mounted with a coverslip using an appropriate mounting medium.

### 2.9. Statistical Analysis

The statistical power of this study was high; GPowerNT 3.1.9.7 software was used to calculate the sample size. The configuration for this calculation was based on a large effect size (0.8), a one-tailed test, and alpha and beta values of 0.1 and 0.9, respectively.

All statistical analyses were performed using GraphPad Prism 8.0.2 software with an alpha level of 0.01 to evaluate normality. Data were cleaned by identifying outliers with the Iterative Grubbs method. A mixed-effects model for repeated measures (with Sidak correction) was used to assess differences in variables such as body weight, fasting glucose and glucose challenge. Pancreatic and adipose tissue weights, along with related indexes, were analyzed using unpaired *t*-tests, Mann–Whitney *U* tests, or Kolmogorov–Smirnov tests, as appropriate. Statistical significance was set at a *p* value < 0.05.

## 3. Results

### 3.1. HCD Induces Hyperglycemia with Higher Insulin and Triglyceride Levels in BALB/c Mice

**Figure 1:**
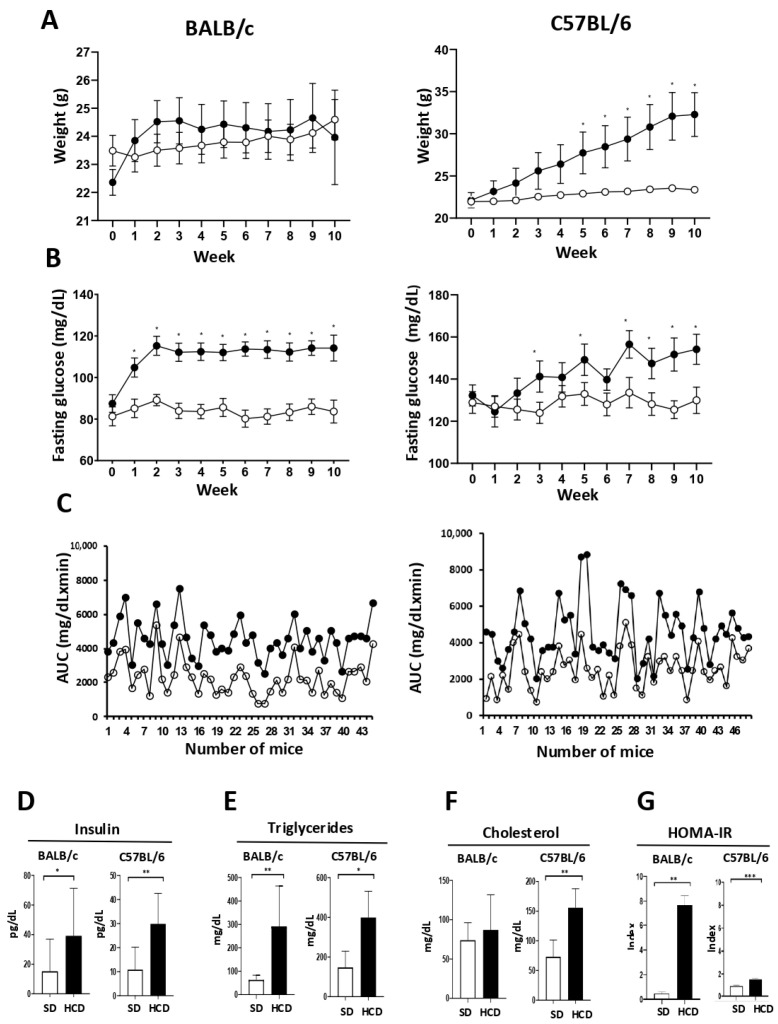
The high-calorie diet (HCD) induces an increase in blood glucose, serum insulin, HOMA-IR index, and triglycerides in serum in BALB/c mice. Both BALB/c and C57BL/6 mice were fed their respective HCD or standard diet (SD) for 10 weeks. Body weight (**A**) and blood glucose (**B**) were measured weekly for each group. * Indicates a statistically significant difference (*p* < 0.05) according to a mixed model test (week factor *p* < 0.0001, diet factor *p* < 0.0001). The graph displays the mean and standard error with n = 24. (**C**) Fasting and post-challenge glycemia in BALB/c and C57BL/6 mice is shown in the area under the curve (AUC), which was determined in 10 weeks by trapezoidal method. To all the graphs, the line with black circles corresponds to animals fed HCD, and the line with white circles corresponds to animals fed SD, statistical differences were determined by U Mann–Whitney test, *p* < 0.0001 to C57BL/6 and by T student *p* < 0.0001 to BALB/c. Insulin (**D**), triglycerides (**E**), and cholesterol (**F**) levels in serum were also measured in these same animals after 10 weeks. (**G**) The HOMA-IR index is shown to both strain-fed SD and HCD. * Indicates *p* < 0.05, ** indicates *p* < 0.01 and *** *p* < 0.001 according to *t*-test (triglycerides BALB/c, cholesterol BALB/c), Kolmogorov–Smirnov (triglycerides and cholesterol C57BL/6) and U Mann–Whitney test (insulin BALB/c and C57BL/6). Bar graphs show mean and standard deviation. C57BL/6 mice were used as the control model for type 2 diabetes mellitus (T2DM).

Mice were monitored weekly for 10 weeks to assess body weight and fasting glucose levels. Insulin, triglycerides, and cholesterol in serum were also measured in mice, but only at the 10-week mark. Fasting glucose levels differed between the two strains: BALB/c mice averaged 85.14 mg/dL ± 2.53, and C57BL/6 mice averaged 128.7 mg/dL ± 3.3. BALB/c mice fed the novel HCD showed significant hyperglycemia starting from week 1 (112.6 mg/dL ± 3.40), continuing through week 10 (116.4 mg/dL ± 5.4), compared to SD controls (85.14 mg/dL ± 2.53) (*p* < 0.05). This finding contrasts with previous reports using standard HFDs, as noted by other authors [19] (Figure 1B). C57BL/6 mice also developed hyperglycemia with HCD (142.9 mg/dL ± 10.34) compared to 128.7 mg/dL ± 3.3 in the SD group, with significance beginning at week 3 (*p* < 0.05) (Figure 1B). Interestingly the body weight in BALB/c mice fed HCD remained unchanged (24.25 g ± 0.67) while C57BL/6 mice exhibited significant weight gain starting at week 5 (27.2 g ± 3.62 on HCD versus 22.8 g ± 5.4 on SD) (*p* < 0.05) (Figure 1A). Notably, C57BL/6 mice fed the BALB/c-specific HCD died within three weeks, and autopsies revealed severe intravascular clotting leading to heart infarction. These damages are attributed to the high caloric content in this strain’s diet. A post-challenge glycemia assay was also conducted at 10 weeks of study, and the results are shown in mg/dL·min, which correspond to the area under the curve (AUC). The AUC of HCD-fed BALB/c mice was significantly higher than that of SD-fed mice (*p* < 0.05) (Figure 1C), indicating that mice with hyperglycemia induced by HCD were unable to efficiently reduce glucose levels over the studied period, unlike SD mice. Similar results were observed in C57BL/6 mice fed HCD and SD, which were used as a model of T2DM (Figure 1C). 

BALB/c strain mice fed with HCD developed hyperglycemia over 10 weeks (116.4 mg/dL ± 5.4). They also had an increase in serum insulin levels (39.09 pg/dL ± 25.94, *p* < 0.05) and serum triglycerides (290.8 mg/dL ± 139.5, *p* < 0.01) at the end of the study compared to mice fed with SD (insulin: 15.16 pg/dL ± 7.85, triglycerides: 63.10 ± 15.99 mg/dL). Similar results were observed in the C57BL/6 strain (Figure 1D,E). Serum cholesterol levels also increased in HCD-fed mice (155.7 mg/dL ± 38.31) compared to SD-fed mice (73.15 mg/dL ± 30.46), but this was only seen in the C57BL/6 mice (Figure 1F). The HOMA-IR index was determined at 10 weeks in the mice. The HOMA index was high (7.68 ± 1.49) in BALB/c mice fed HCD compared to (0.48 ± 0.27) in mice fed SD of the same strain (*p* < 0.01). Similar results were obtained in C57BL/6 mice (*p* < 0.001) (Figure 1G). All these findings suggest that BALB/c mice, like C57BL/6 mice, presented insulin resistance and developed induced T2DM with the studied HCD.

### 3.2. Intake of Food and Sucrose-Supplemented Water

Table 3 displays the average food and water intake of BALB/c and C57BL/6 mice fed either an SD or an HCD for 10 weeks and shows the total Kcal intake of each diet. Both strains ate more solid food on the SD (to BALB/c 75.14 g, ±2.42; to C57BL/6 108.3 g, ±2.41 respectively) compared to the HCD (to BALB/c 41.89 g, ±4.49; to C57BL/6, ± 71.45, 11.11 respectively), likely because the HCD was less palatable. However, water intake with sucrose was significantly higher in C57BL/6 mice (217.2 mL, ±20.9) compared to BALB/c mice (71.57 mL, ±11.85) and to animals that drank water without sucrose (to BALB/c, 77.64 g, ±3.38; to C57BL/6 131.2 g, ±5.9 respectively). This indicates that C57BL/6 mice have a stronger preference for sucrose, which may increase their risk of developing T2DM compared to BALB/c mice.

Although food consumption was lower in mice fed the HCD, the total caloric intake was higher in both strains because of the additional calories from sucrose-supplemented water. On average, BALB/c mice consumed 297.04 kcal, ±20.92 on the HCD compared to 251.72 kcal, ±8.13 on the SD. In contrast, C57BL/6 mice consumed 362.43 kcal, ±8.3 on the SD and 482.54 kcal, 51.14 on the HCD. Overall, C57BL/6 mice ingested more total calories than BALB/c mice, likely due to an inherently higher appetite.

The results presented in Table 3 and Figure 1 demonstrate that both BALB/c and C57BL/6 mice developed elevated serum glucose, triglycerides, and insulin levels—key indicators of T2DM—when exposed to diets with high caloric content.

### 3.3. The New HCD Induced Adipose and Pancreatic Tissue Alterations

**Figure 2:**
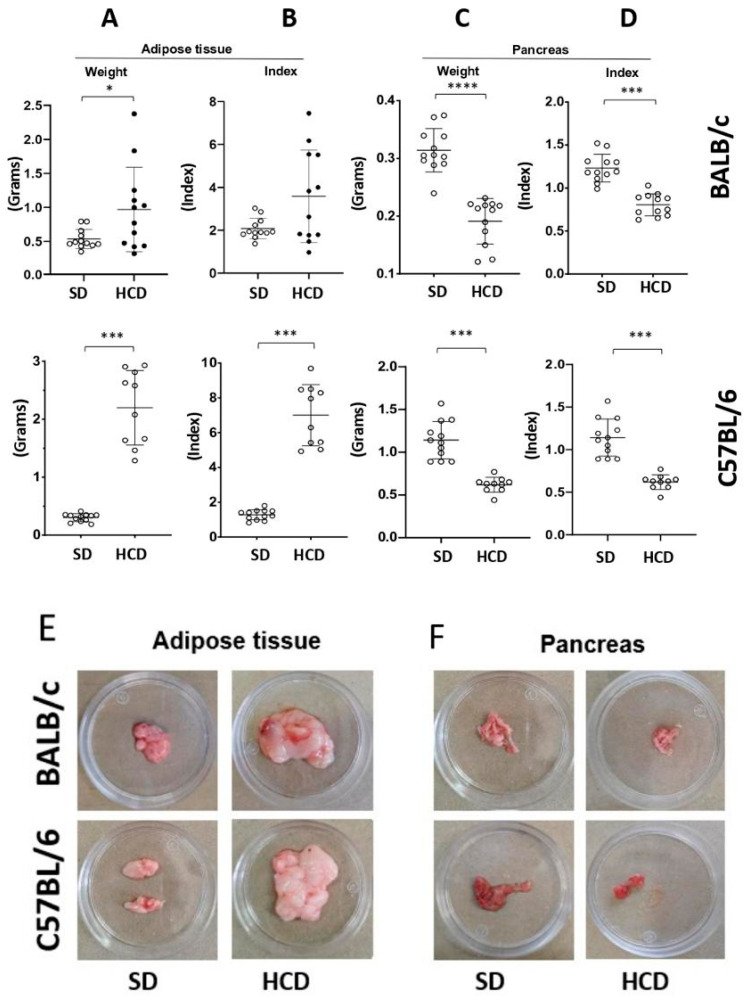
The new high-calorie diet (HCD) induced alterations in the mass of adipose and pancreatic tissues. BALB/c and C57BL/6 mice were fed their corresponding HCD or standard diet (SD). After 10 weeks, adipose and pancreatic tissues were collected to determine the weight of adipose tissues (**A**) and of pancreas (**C**), as well as the index of adipose tissue (**B**) and pancreatic tissue (**D**) in both mouse strains. (**E**) shows images of adipose tissues, and in (**F**), the pancreas from both strains fed with HCD and SD. * Indicates statistically significant difference (* *p* < 0.05 *** *p* < 0.001 **** *p* < 0.0001) by Student’s *t*-test (adipose weight BALB/c and C57BL/6, pancreas weight BALB/c and C57BL/6, pancreatic index BALB/c and C57BL/6, adiposity index C57BL/6), Kolmogorov–Smirnov test (adiposity index BALB/c).

In week 10, all the animals in the study were euthanized, and adipose and pancreatic tissues were analyzed. During this period, BALB/c mice fed with HCDs showed significant increases in visceral fat weight (0.96 g ± 0.62 compared to HCD and 0.53 g ± 0.14 compared to SD), (*p* < 0.05). Similar results were observed in the susceptible C57BL/6 mice, but with more pronounced differences compared to the mice fed with SD (2.19 g ± 0.64 compared to HCD and 0.30 g ± 0.06 compared to SD) (*p* < 0.001) (Figure 2A,E). The adipose index was also higher in both mouse strains fed with HCD (to BALB/c 3.58 ± 2.16; to C57BL/6 7.0 ± 1.7) compared to animals fed with SD (to BALB/c 2.08 ± 0.48; to C57BL/6 1.27 ± 0.29); the difference was significant only in C57BL/6 mice (*p* < 0.001) (Figure 2B). In contrast, both BALB/c (0.19 g ± 0.03 compared to HCD and 0.31 g ± 0.03 compared to SD) and C57BL/6 (0.19 g ± 0.03 compared to HCD and 0.26 g ± 0.05 compared to SD) mice experienced significantly reduced pancreatic weight (*p* < 0.0001 for BALB/c and *p* < 0.001 for C57BL/6) when fed with HCD (Figure 2C,F), which was also reflected in a decrease in the pancreatic index in the same animals fed with HCD. The pancreatic index for BALB/c fed HCD was approximately 0.8 ± 0.12, while for those fed SD it was 1.23 ± 0.16. For C57BL/6, the fed HCD was 0.61 ± 0.08, and the fed SD was 1.14 ± 0.21 (*p* < 0.0001) (Figure 2D). These results suggest tissue atrophy and pancreatic dysfunction.

### 3.4. Histopathological Findings in the Pancreas and Liver of HCD-Fed Mice

**Figure 3:**
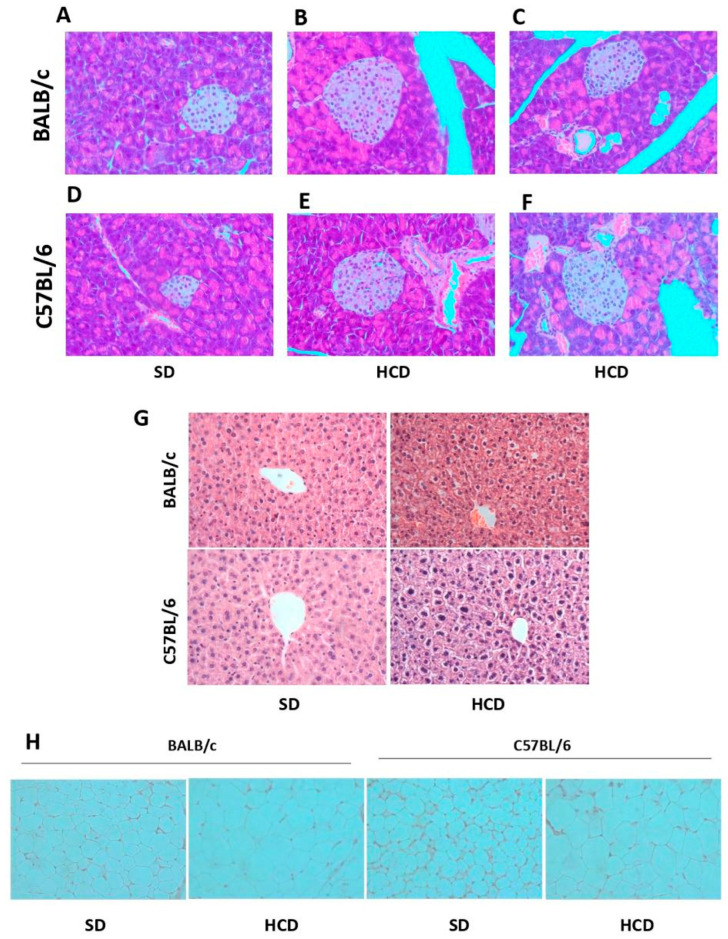
The new high-calorie diet (HCD) altered the histological structure of pancreatic islets, liver, and adipose tissues in BALB/c mice. Representative histological sections stained with hematoxylin and eosin (H&E) at 40× magnification is shown. (**A**–**C**) correspond to pancreatic tissue from BALB/c mice, while (**D**–**F**) correspond to pancreatic tissue from C57BL/6 mice, both of which were fed either an HCD or an SD. Additionally, liver tissue (**G**) and adipose tissue (**H**) sections from both strains under the same dietary conditions are presented.

H&E staining revealed significant disorganization in the architecture of pancreatic islets in BALB/c mice fed with HCD, as well as in the pancreatic islets of C57BL/6 mice fed with the corresponding HCD. Both strains exhibited cellular pyknosis and a disorganized cell chain (Figure 3B,C), which was not observed in the pancreatic islets of mice fed with SD, where the islet cell chains are well organized. Several fatty infiltrates were observed in the non-endocrine tissues of the pancreas in HCD-fed BALB/c mice (Figure 3C). As shown in Figure 3A–C, islets of HCD-fed BALB/c and HCD-fed C57BL/6 mice appeared enlarged compared to SD-fed mice. Additionally, a common histopathological characteristic of humans and animals with T2DM is hepatic steatosis and changes in adipose tissue histology that are hallmark of non-alcoholic fatty liver disease (NAFLD) which is a comorbidity in T2DM [32]. Liver sections from BALB/c mice fed with HCD demonstrated pronounced steatosis, characterized by large lipid inclusions. Similar results were observed in liver sections from C57BL/6 mice fed with the corresponding HCD. This hepatic steatosis was not found in animals fed with SD (Figure 3G). Fatty tissues from BALB/c and C57BL/6 mice fed with HCD showed hypertrophic adipocytes, which were not observed in animals fed with SD (Figure 3H). All these data support insulin resistance and metabolic dysfunction, confirming that BALB/c mice fed with HCD in this work can develop T2DM.

## 4. Discussion

Commercial HFDs are costly, have a short half-life, and are not optimized for all strains. Our study introduces strain-specific, affordable formulations that successfully induce T2DM in BALB/c and C57BL/6 mice. Contrary to previous beliefs, BALB/c mice can develop hyperglycemia and T2DM when exposed to a sufficiently calorie-dense diet. The lethal response of C57BL/6 mice to the BALB/c-specific diet highlights strain-specific metabolic tolerances [27,28].

Our findings align with previous research, which shows that diet-induced T2DM can be induced in BALB/c mice when combined with streptozotocin. However, our model achieves this without the use of pharmacological agents [33,34,35].

Our model shows that BALB/c mice can develop hyperglycemia along with high levels of insulin and triglycerides in serum, hepatic steatosis and adipocyte hypertrophy. These changes can lead to T2DM, as seen in the HCD-fed C57BL/6 mouse strain, but this depends on the formulation of the HCD. Furthermore, the high AUC and HOMA index values suggest that BALB/c mice developed insulin resistance when fed HCD.

Other studies using high-fat diets (e.g., 60% of calories from fat) also reported a delayed onset of hyperglycemia in BALB/c mice, suggesting that dietary composition is a key factor. Researchers have shown that, with the HCD used, BALB/c mice developed hyperglycemia until the 9th week, whereas in C57BL/6 mice hyperglycemia appeared by the 4th week and persisted until 10 weeks [27,28]. Using our specific HCD formulations for both strains, BALB/c mice exhibited elevated blood glucose levels from the first week of treatment, whereas C57BL/6 mice only experienced high blood glucose after three weeks. In the 10th week, both strains fed the specific HCD showed increased insulin and triglyceride levels. It is important to note that the fat percentage in the diet for BALB/c mice was higher than that in the diet used for C57BL/6 mice to induce hyperglycemia. These findings suggest that the fat content in the HCD is a crucial factor in inducing hyperglycemia in BALB/c mice, which correlates with the elevated serum triglyceride levels observed in these animals. Most previous reports used the same HCD formulation for both strains, which may explain why BALB/c mice do not present T2DM. Notably, when the HCD formulated for BALB/c mice was given to C57BL/6 mice, most died within 3 weeks.

Although the increase in visceral fat in HCD-fed BALB/c mice was not as significant as in HCD-fed C57BL/6 mice, the high triglyceride levels in HCD-fed BALB/c mice and the hypertrophy observed in adipocytes, which indicates adipose tissue dysfunction, suggest that HCD-fed BALB/c mice can accumulate visceral fat despite not gaining weight. Then, the fat in the visceral tissues triggers an inflammatory process, which can lead to insulin resistance and T2DM. The post-challenge glycemia and HOMA-IR results in our mice suggest they had insulin resistance. Proinflammatory mediators, such as interleukin IL-6, (IL)-1β, and TNF-α, are known to be involved in insulin resistance and have been detected in peripheral adipose tissue and associated with the apoptosis of pancreatic β-cells. Recently, other new members of the IL-1 family, such as IL-36, IL-37, and IL-38, have been more strongly associated with T2DM [36,37].

On the other hand, specific HCD formulations in BALB/c mice induced pancreatic changes, including reduced size, hepatic steatosis, and adipose tissue alterations similar to those observed in C57BL/6 mice, confirming that BALB/c mice can also develop T2DM. Other authors have reported these pathological features in diet-induced T2DM mouse models, which are promoted by proinflammatory cytokines produced by recruited circulating macrophages that drive fatty liver development and contribute to impaired hepatic insulin sensitivity [27,30,31,38,39]. In humans, NAFLD is a common comorbidity of T2DM, and hepatic steatosis is a hallmark of this disease. In a meta-analysis of 1,832,125 patients, a high prevalence of NAFLD in people with T2DM was found, suggesting that hepatic steatosis is an essential factor in the diagnosis of diabetes [32,40]. In addition, the increase in serum insulin observed in our BALB/c and C57BL/6 mice fed HCD has also been reported by other authors in C57BL/6 mice with diet-induced T2DM [27,30,31]. This increase corresponds to a compensatory hyperinsulinemia in response to hyperglycemia.

Show an animal model of diabetes in BALB/c mice, which, in the majority of works, is present as having low or null susceptibility to develop diabetes; it is essential, because with our model, we can study the molecules involved in the development of diabetes. Some studies have reported on the susceptibility of BALB/c. A study examining lipid intermediates in different mouse strains concluded that the relative protection of BALB/c mice against diet-induced T2DM is not due to differences in the total amounts of specific lipids such as triacylglycerol, diacylglycerol, ceramide, sphingomyelin, phosphatidylcholine, and phosphatidylethanolamine, which are linked to T2DM [41].

The report by Salazar-León in 2019 associates inflammasome molecules with BALB/c’s susceptibility to obesity. Several reports have shown that genetic predisposition plays a significant role in T2DM, while dietary factors also influence its onset and severity. Differences in inflammasome gene expression (e.g., Nlrp3, Nlrp1b, Nod2) between strains may contribute to differences in susceptibility to obesity [32]. Notably, obesity and blood chemistry values of Nod2−/− BALB/c mice fed a standard diet were similar to those of wild-type (WT) BALB/c mice fed the same diet, while those of the mice fed with an HCD presented different values between Nod2−/− and wild-type [31]. These results and our findings indicate that genetics plays a crucial role; however, environmental factors, such as dietary composition and caloric intake, are also significant in the development of metabolic disorders, including T2DM.

Several studies could be conducted using the model we showed, such as comparing the profile of proinflammatory cytokines (IL-36) or studying the metabolism of pancreatic beta-cells in mice with low and high susceptibility, which is important to investigate. New drugs could also be tested with this model.

## 5. Conclusions

BALB/c mice are traditionally considered to have low susceptibility to develop T2DM when exposed to a specific HCD formulation. This underscores the dual importance of genetic and dietary factors in T2DM pathogenesis and establishes a new, practical model for further research.

## Figures and Tables

**Table 3:** Food and water intake by BALB/c and C57BL/6 mice fed with SD or HCD, and the total Kcal intake by each strain.

	BALB/c				C57BL/6			
Diet	SD		HCD		SD		HCD	
	Intake	KCal	Intake	KCal	Intake	KCal	Intake	KCal
Food (g/box/week)	75.14 ± 2.42	251.72 ± 8.13	41.89 ± 4.49	204.5 ± 20.8	108.3 ± 2.41	362.44 ± 8.3	71.45 ± 11.11	308.79 ± 47.19
Water (mL/box/week)	77.64 ± 3.38	0	71.57 ± 11.85 (sucrose 30%)	92.19 ± 22.34	131.2 ± 5.9	0	217.2 ± 20.9 (sucrose 20%)	173.76 ± 16.72
Total KCal		251.72 ± 8.13 *		297.04 ± 20.92 *		362.43 ± 8.3 †		482.54 ± 51.14 †

The data presented correspond to the average food and water intake over a 10-week period for the mice included in the study. The caloric values of the diets were calculated based on the information shown in Table 2: each gram of SD provided 3.35 kcal, while the HCD provided 4.89 kcal for BALB/c mice and 4.31 kcal for C57BL/6 mice. The caloric contribution from sucrose-supplemented water was estimated assuming that each milliliter of 30% sucrose solution provided 1.2 kcal and each milliliter of 20% sucrose solution provided 0.8 kcal. Each cage contained three mice. Symbols indicate statistically significant differences between SD and HCD, BALB/C * (*p* < 0.0001) and SD and HCD C57BL/6 † (*p* < 0.0001).

## Data Availability

The original contributions presented in this study are included in the article. Further inquiries can be directed to the corresponding authors.

## Abbreviations

The following abbreviations are used in this manuscript:

BMI	Body mass index
ENCB-IPN	Escuela Nacional de Ciencias Biológicas del Instituto Politécnico Nacional
GK	Goto-Kakizaki rats
H&E	Hematoxylin and eosin
HCDs	High-calorie diets
HFD	High-fat diet
NOD	Non-obese diabetic mice
SD	Standard diet
T2DM	Type 2 diabetes mellitus
WT	Wild-type
ZDF	Zuker diabetic fatty
